# Somatic symptoms, psychological distress and trauma after disasters: lessons from the 2014 Hazelwood mine fire and 2019–20 Black Summer bushfires

**DOI:** 10.1186/s12889-023-16501-1

**Published:** 2023-08-18

**Authors:** Caroline X. Gao, Jana Menssink, Timothy C. H. Campbell, Catherine L. Smith, Jillian F. Ikin, Tyler Lane, Michael J. Abramson, Matthew Carroll

**Affiliations:** 1 School of Public Health and Preventive Medicine, Monash University, 553 St Kilda Road, Melbourne, VIC 3004 Australia; 2 Centre for Youth Mental Health, The University of Melbourne, 35 Poplar Road, Parkville, VIC 3052 Australia; 3 Orygen, 35 Poplar Road, Parkville, VIC 3052 Australia; 4 Monash Rural Health, Monash University, Northways Road, Churchill, VIC 3842 Australia

**Keywords:** General psychological distress, Posttraumatic stress, Repeated disaster exposure, Smoke exposure, Somatic symptoms, Wildfires

## Abstract

**Background:**

Wildfires cause significant physical and mental ill-health. How physical and mental symptoms interact following wildfire smoke exposure is unclear, particularly in the context of repeated exposures. In this cross-sectional study we investigated how posttraumatic stress and general psychological distress associated with somatic symptoms in a community exposed to multiple smoke events.

**Methods:**

A random weighted sample of 709 adults exposed to smoke during the 2014 Hazelwood coal mine fire in south-eastern Australia completed a survey in 2020. The survey coincided with the Black Summer wildfires that caused a similar period of smoke haze in the region. Participants self-reported somatic symptoms (PHQ-15) and mine fire-related posttraumatic stress (IES-R) experienced over the previous week, general psychological distress (K10) experienced over the previous four weeks, lifetime health diagnoses and demographic information. Associations between posttraumatic stress, general psychological distress, and each PHQ-15 somatic symptom were analysed using ordinal logistic regression models.

**Results:**

Overall, 36.2% of participants reported moderate- or high-level somatic symptomology. The most frequent somatic symptoms were fatigue, limb pain, trouble sleeping, back pain, headaches, and shortness of breath. After controlling for confounding factors, general psychological distress and posttraumatic stress were independently associated with all somatic symptoms (except menstrual problems in females for posttraumatic stress).

**Conclusions:**

Results highlight the high prevalence of somatic symptoms and their association with general psychological distress and posttraumatic stress within a community in the midst of a second large-scale smoke event. It is essential that healthcare providers and public health authorities consider the interconnections of these conditions when supporting communities affected by climate-related disasters.

**Supplementary Information:**

The online version contains supplementary material available at 10.1186/s12889-023-16501-1.

## Introduction

In the context of climate change, weather-related disasters have become increasingly common, causing substantial health, social and economic impacts [1]. The proliferation of catastrophic wildfire events is perhaps the most salient example of the consequences of global warming [2, 3]. With fire seasons getting longer and fire-prone areas increasing in number and size in many countries, wildfires are arguably the most frequently occurring type of environmental disaster in the world today, impacting an ever-growing number of people [4].

Wildfires pose a unique set of dangers and challenges to those they impact. They can quickly escalate into extremely large and complex situations that are unpredictable and difficult to contain [5]. Hence, they can become protracted public emergencies involving long periods of hazard, disruption, relocation, and recovery [6]. Wildfires emit large volumes of air-polluting smoke that can disperse to places far distant from the fire’s epicentre, with the potential to adversely affect large numbers of people [7]. Wildfires, particularly those of extended scale and duration, cause significant physical and mental ill-health [8]. Globally, between 2000 and 2016, over 33,000 deaths were attributable to wildfire smoke exposure [9]. Between 2013 and 2018, wildfire smoke in Canada was estimated to have a yearly economic burden of CDN$410 M–$1.8B for acute health impacts and CDN$4.3B–$19B for chronic health impacts [10]. The psychological sequelae of wildfire exposure may persist, increasing the risk of mental disorders for years after the event [11].

An important, yet sometimes overlooked, topic is the interaction between physical and mental health symptoms following climate-related disasters. Psychopathology, particularly posttraumatic stress symptomatology (PTSS) and posttraumatic stress disorder (PTSD), has been linked with a range of somatic (physical) symptoms, for example, persistent pain, fatigue, shortness of breath and gastrointestinal problems [12–14]. Somatic symptoms are highly prevalent among disaster-exposed communities (e.g., man-made disasters, earthquakes) [15–17]. Simultaneously, physical conditions can exacerbate psychological distress and illness [18]. However, the association between psychological distress and somatic symptoms following repeated large-scale smoke events is largely unknown.

In 2014, the Hazelwood coal mine in eastern Victoria, Australia, was ignited by wildfires and burned for 45 days, covering surrounding areas in smoke. The mine fire was one of the worst pollution events recorded in Victoria [19], prompting considerable community concern regarding short and long-term health impacts. The Hazelwood Health Study (HHS; www.hazelwoodhealthstudy.org.au) was established to evaluate the health impacts arising from the mine fire. The HHS Adult Survey established and surveyed an adult population cohort in May 2016-February 2017 [20]. To further investigate longer-term psychological impacts, a follow-up Mental Health and Wellbeing Survey was conducted with a subsample of the cohort. The timing of the follow-up survey happened to coincide with the Black Summer wildfires (September 2019-March 2020). The local region was not directly impacted by fire activity during the Black Summer, however, plumes of smoke from fires burning nearby and across south-eastern Australia were distributed into the region and generated hazardous pollution levels during the survey period [21]. This analysis explored the role of mine fire-related posttraumatic stress and general psychological distress in the concurrent presentation of somatic symptoms during the Black Summer event.

## Methods

A sub-sample of HHS Adult Survey participants was randomly selected and invited to participate in the Mental Health and Wellbeing Survey between December 2019 and March 2020. Participants (*n* = 709 out of 1,512 invitations) were residents in Morwell at the time of the mine fire, the town closest to the mine and, consequently, the most exposed to smoke [19]. Participants were aged at least 18 years at the time of the event.

Somatic symptoms experienced during the previous week were measured using the Patient Health Questionnaire (PHQ-15; scoring from 0 to 30). The PHQ-15 is a valid and reliable questionnaire to detect and monitor changes in somatic symptoms, with cut-off points of 5, 10, and 15 representing low, medium, and high somatic symptom severity respectively [22]. Posttraumatic stress symptoms (PTSS) experienced in the previous week, specifically associated with the 2014 Hazelwood coal mine fire, were measured using the Impact of Event Scale-Revised (IES-R; scoring from 0 to 88) [23]. The IES-R is a clinically validated measure of PTSS severity associated with a traumatic event, with scores of ≥ 24 considered to be of clinical concern [24]. General psychological distress experienced during the previous four weeks was measured using the Kessler Psychological Distress Scale (K10; scoring from 10 to 50). Scores of ≥ 22 indicate ‘high’ to ‘very high’ distress levels [25]. The Mental Health and Wellbeing Survey also collected information about doctor-diagnosed mental health conditions (anxiety, depression and other) and employment status. Information about diagnosed physical diseases (including cardiovascular diseases, asthma, COPD, cancer and diabetes) and mental health conditions, age, gender, highest educational level, and smoking status was sourced from the earlier Adult Survey. All measures were self-reported.

The survey was powered to detect a 2-point change in IES-R cores of participants from round 1 survey to round 2 survey (assuming a standard deviation of 4 points). It was determined that approximately 450 participants would be needed to detect the effect with at least 90% power using mixed-effects regression analysis. A detailed description of the recruitment methods, study design and instruments is published elsewhere [26].

Descriptive statistics were used to explore differences between participants presenting with different levels of somatic symptom severity. To evaluate how somatic symptomology, psychological distress and mine fire-related posttraumatic stress overlapped, the prevalence of co-occurring conditions was mapped using a Venn diagram. Associations between individual items across the three instruments were evaluated in a psychometric network analysis (illustrated using a Multidimensional scaling plot of pairwise polychoric correlations) [27].

To evaluate the independent associations between posttraumatic stress, general psychological distress and individual somatic symptoms, ordinal logistic regression modelling was carried out for each PHQ-15 item, essentially creating 15 outcome variables with IES-R and K10 total scores treated as risk factors. Odds Ratios (ORs) and 95% Confidence Intervals (95% CIs) were estimated to determine the likelihood of somatic symptomology for each one standard deviation (SD) increase in IES-R and K10 total scores. Potential confounders including age, gender, education and employment status, smoking, and self-reported physical and mental health conditions were controlled for. IES-R and K10 total scores were standardised to enable easier comparison.

A range of sensitivity analyses were carried out to validate the findings, including: (1) controlling for IES-R and K10 in separate models; (2) removal of potential overlapping items in total scores; and (3) similar linear regression models, with PHQ-15 total score as the outcome (including and excluding overlapping items). All analyses were conducted using R version 4.2.2 (2022–10-31). Missing data were imputed using five imputed datasets and regression results were pooled using Rubin’s rules [28].

## Results

Descriptive statistics for the sample, and for somatic symptom severity sub-groups, are provided in Table 1. Detailed comparisons between survey responders and non-responders are published elsewhere [26]. The sample had a slightly higher proportion of females than males and a median age of 53 years (IQR 37–66). Over a third (36.2%) of participants reported a medium or high level of somatic symptoms (PHQ-15, median: 7; IQR: 3–12). Somatic symptom severity was positively associated with unemployment or inability to work, smoking, and physical or mental health conditions, as well as general psychological distress and mine fire-related posttraumatic stress.

**Table 1:** Participant descriptive characteristics by somatic symptom severity

			**Somatic symptom severity group**				
**Characteristic**		**Total *N* = 709**	**Minimal (0–4) *n* = 239**	**Low (5–9) *n* = 205**	**Medium (10–14) *n* = 142**	**High (15–30) *n* = 110**	***p*-value**
Age	median (IQR)	53 (37, 66)	51 (36, 65)	54 (37, 67)	58 (43, 71)	49 (36, 60)	0.005
Gender							< 0.001
Female	n (%)	389 (55%)	105 (44%)	125 (61%)	87 (62%)	64 (58%)	
Male	n (%)	319 (45%)	134 (56%)	80 (39%)	54 (38%)	46 (42%)	
Education^a^							0.4
Year 12 or under	n (%)	300 (43%)	92 (39%)	89 (45%)	63 (46%)	50 (46%)	
Post-secondary	n (%)	393 (57%)	144 (61%)	109 (55%)	75 (54%)	58 (54%)	
Employment^a^							< 0.001
Paid employment	n (%)	381 (55%)	153 (65%)	114 (57%)	62 (44%)	46 (42%)	
Other (student; volunteer; home-duties; retired)	n (%)	229 (33%)	64 (27%)	73 (36%)	59 (42%)	32 (29%)	
Unemployed or unable to work	n (%)	89 (13%)	20 (8.4%)	14 (7.0%)	19 (14%)	31 (28%)	
Smoking^a^							0.003
Non-smoker	n (%)	416 (59%)	157 (66%)	117 (58%)	81 (57%)	52 (47%)	
Former smoker	n (%)	183 (26%)	57 (24%)	59 (29%)	34 (24%)	30 (27%)	
Current smoker	n (%)	107 (15%)	24 (10%)	27 (13%)	27 (19%)	28 (25%)	
Diagnosed with a physical disorder^a^	n (%)	369 (52%)	99 (41%)	109 (53%)	76 (54%)	79 (72%)	< 0.001
Diagnosed with a mental disorder^b^	n (%)	308 (43%)	58 (24%)	87 (42%)	83 (58%)	76 (69%)	< 0.001
IES-R total score (scores 0–88)	median (IQR)	5 (0, 17)	1 (0, 6)	4 (0, 14)	12 (4, 24)	26 (11, 46)	< 0.001
K10 total score (scores 10–50)	median (IQR)	16 (12, 22)	12 (10, 14)	15 (13, 20)	19 (15, 25)	30 (23, 34)	< 0.001

Statistics presented are median (IQR) and count (percentage). Missing data include 1 record for gender, 16 records for education, 10 records for employment, 3 records for smoking, 22 records for IES-R, 19 records for K10 and 13 records for PHQ-15 severity group

^a^Only measured in the Adult Survey

^b^Measured in both the Adult Survey and the follow-up Mental Health and Wellbeing Survey with the data combined to improve accuracy

**Fig. 1:**
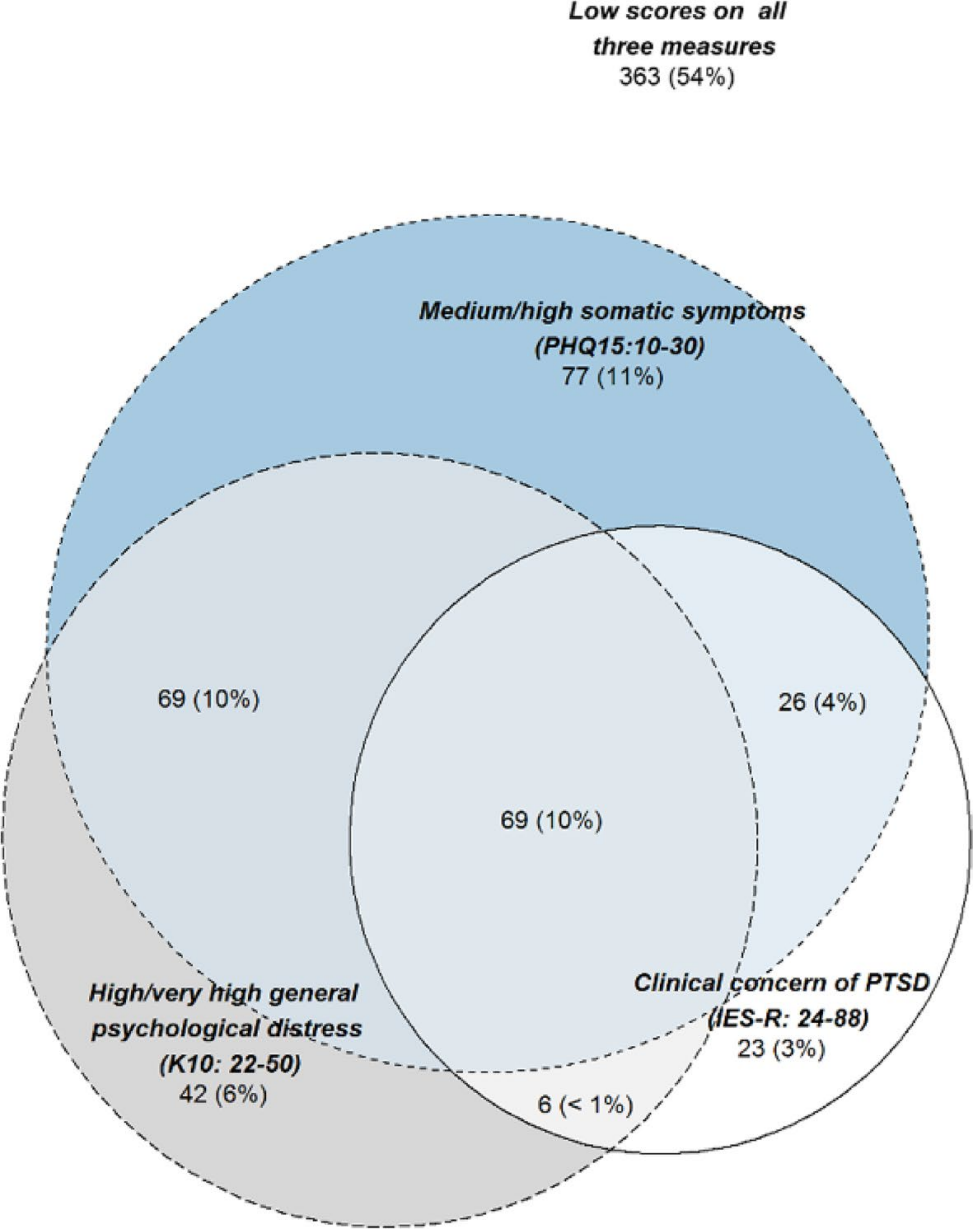
Venn diagram showing the intersection of general psychological distress (measured by K10), posttraumatic stress related to the Hazelwood mine fire (measured by IES-R) and somatic symptoms (measured by PHQ-15) shared between participants. Note. *N* = 675 with non-missing information from all three measures

The distributions of K10 and IES-R total scores across individual PHQ-15 items were evaluated using boxplots (see Supplementary Figures S1 and S2). General psychological distress and mine fire-related posttraumatic stress were consistently associated with higher symptom severity across all PHQ-15 items. Figure 1 presents a Venn diagram illustrating overlaps between higher levels of somatic symptomology, psychological distress and mine fire-related posttraumatic stress. Approximately a quarter of participants presented with two or more symptomologies exceeding cut-off scores across these three constructs.

**Fig. 2:**
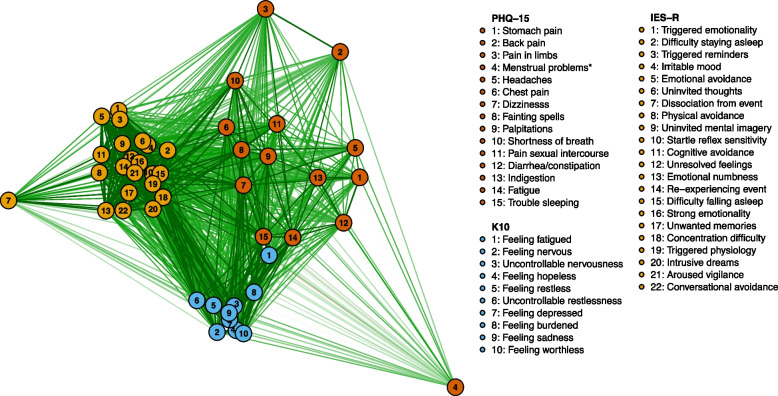
Psychometric network plot of all items from the PHQ-15, K10, and IES-R measures. Note. Distances between nodes were estimated based on Multidimensional Scaling (MDS) of pairwise polychoric correlations; closer proximity and darker coloured lines between nodes represent stronger correlations

As shown in the network plot (Fig. 2), most items in the K10 and IES-R are grouped closely together. The inter-item correlations for PHQ-15 were lower and the ‘fatigued’ item (Q14) correlated highly with the K10 ‘feeling fatigued’ item (Q1; polychoric correlations of 0.74; see Figure S3). The PHQ-15 ‘trouble sleeping’ item was also highly correlated with K10 items and some IES-R items (see Figure S3).

**Fig. 3:**
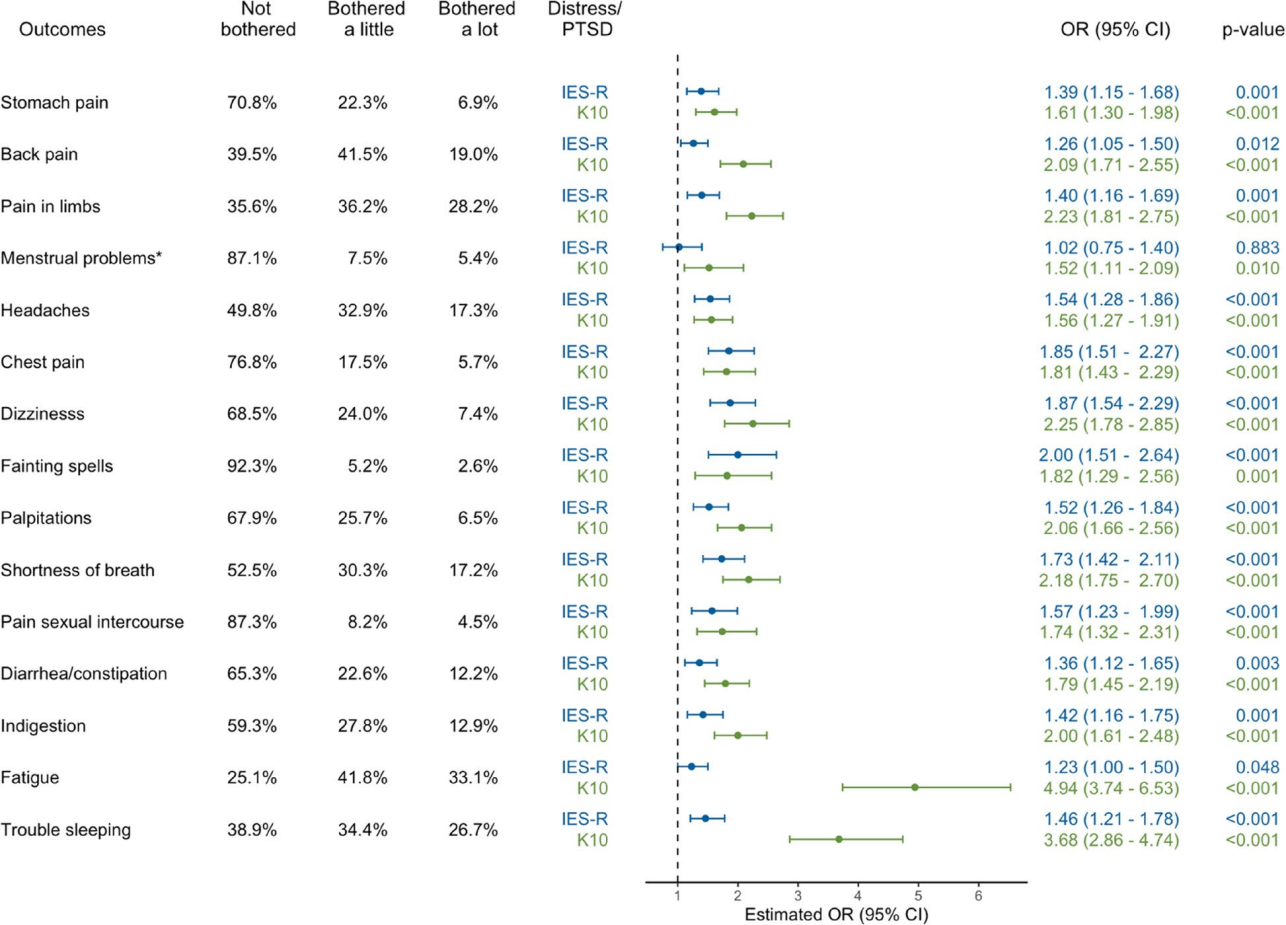
Response patterns to individual somatic symptoms and estimated OR (95% CI) associated with a one standard deviation (SD) increase in IES-R (SD = 16.2) and K10 (SD = 8.2) scores. Note: for each somatic symptom, one imputed ordinal logistic regression model was used including both IES-R and K10 as risk factors and controlling for confounders including age, gender (*except for menstrual problems which were evaluated in females only), education, employment, smoking status, diagnosed mental health conditions, and diagnosed physical health conditions

Figure 3 presents the response patterns of each individual somatic symptom and estimated ORs and 95% CIs. The most prevalent somatic symptoms were fatigue, limb pain, trouble sleeping, back pain, headaches and shortness of breath. After controlling for confounding factors, K10 and IES-R scores were each independently positively associated with all somatic symptoms, except menstrual problems in females for the IES-R. Fatigue and trouble sleeping were the somatic symptoms most strongly associated with general psychological distress, with a one SD increase in K10 score increasing odds of being bothered by these symptoms by 390% (OR 4.94; 95% CI: 3.74–6.53) and 268% (OR:3.68; 95% CI: 2.86–4.74) respectively. For other somatic symptoms, a one SD increase in IES-R score was associated with a 23–100% increase in the odds of symptomology and a one SD increase in K10 score was associated with a 57–126% increase in odds.

Interestingly, sensitivity analysis indicated that these effect sizes were comparable with models that included K10 and IES-R separately (see Figure S4). This suggests that only a small fraction of the variations in somatic symptomology were explained by features shared between general psychological distress and posttraumatic stress, and that these two conditions had additive effects. Sensitivity analysis excluding the overlapping item of `feeling fatigued’ in K10 showed similar results (see Figure S5) compared with Fig. 3, which suggested that these shared features were not due to overlapping items.

K10 and IES-R scores were each also independently associated with PHQ-15 total scores in linear regression models (see Table S1), with K10 scores found to have comparatively larger effects, which were predominately associated with fatigue and trouble sleeping. Sensitivity analysis excluding three highly correlated items (`feeling fatigued’ in K10; ‘fatigue’ and ‘trouble sleeping’ in PHQ-15) suggested slightly more even contributions of psychological distress and mine fire-related posttraumatic stress to the overall level of somatic symptoms (see Table S2).

## Discussion

Over a third of participants previously exposed to smoke during the 2014 Hazelwood mine fire reported medium to high-severity somatic symptoms during the 2019–20 Black Summer. General psychological distress and mine fire-related posttraumatic stress, simultaneously measured during Black Summer, had an additive association, increasing the severity of a range of somatic symptoms. These results suggest a widespread prevalence of somatic symptoms and links with psychological distress and trauma-related symptoms in a climate disaster-exposed community during a later, similar, event.

There is a growing body of literature demonstrating bidirectional associations between physical and mental ill-health [18]. The link is particularly strong between somatic symptoms and PTSS/PTSD [12, 13], with a range of potential pathophysiological mechanisms implicated, such as suppressed neuroendocrine and immune functions, direct impacts on the central nervous system, or hypervigilance-related somatosensory amplification [13, 29, 30]. The association between somatic symptoms and trauma exposure has been reported following disasters such as earthquakes and hurricanes [15]. General psychological distress was also associated with higher rates of self-reported respiratory symptoms in the earlier Hazelwood Health Study Adult Survey [31]. The present findings suggest that similar associations with psychological distress exist for other physical symptoms and that this is an area of concern following recurring smoke exposure from wildfires.

The observed high prevalence of somatic symptoms, general psychological distress and mine fire-related posttraumatic stress, as well as their strong internal link, were likely associated with participants’ re-exposure to smoke during the 2019–20 Black Summer. Participants reported higher levels of mine fire-related posttraumatic stress at this time-point compared with levels they reported in 2016–17 [26]. Re-exposure to a large-scale smoke event may have simultaneously increased participants’ levels of general distress, triggered trauma responses related to the previous mine fire, and caused physical ill-health (e.g., respiratory conditions). Since individual-level smoke exposure during Black Summer was not measured, its effects could not be directly assessed. However, as a vast majority of participants were living in the same regional town and the source of the smoke was geographically distant, it is likely that smoke exposure levels during Black Summer were reasonably equivalent between participants.

General psychological distress was more strongly associated with somatic symptomology compared with mine fire-related posttraumatic stress. This is likely to have been driven by strong associations between low energy and sleeping problems with psychological distress, which might arguably be tautological, as both symptoms are part of the DSM-5 diagnostic criteria for anxiety and depressive disorders [32].

## Conclusions

The psychological and direct physical health (e.g., respiratory health) impacts of wildfires are receiving increasing attention [8] as are public health responses, including integrating emergency and health service provision, government subsidised psychological services guidance on wearing facemasks, and raising public awareness about health risks in such circumstances [33]. However, the impacts on non-disease specific somatic symptoms remain under-recognised and under-treated.

It is essential that health care professionals who treat patients with physical health complaints in wildfire-affected areas use a biopsychosocial approach sensitive to interconnections between trauma, physical and mental health. The presentation of somatic symptoms often makes it more difficult for primary care clinicians to identify underlying mental health issues [14, 34]. Individuals with PTSS frequently present with physical health complaints prior to seeking help for psychological issues [35]. Therefore, in communities that have been impacted by wildfires or other kinds of large-scale disaster events, it is important to screen and monitor for PTSS and psychological distress among people presenting with physical health complaints to ensure unmet care needs are identified and addressed.

Limb pain, back pain and headaches were among the most frequently reported somatic symptoms in this study. The high level of comorbidity between persistent pain and PTSS highlights the importance of screening and monitoring for both in tandem following wildfires. Persistent pain is a major public health issue that continues to be sub-optimally managed; delayed referrals to multidisciplinary pain clinics, the gold standard treatment for non-cancer pain, often taking place years after the onset of pain [36]. This study specifically highlights the need for better funding and referral pathways to multidisciplinary pain management and care in wildfire-impacted communities. As more climate change-related disasters are anticipated [37], additional health supports are needed to better facilitate diagnosis, referral and treatment in impacted communities.

### Strengths, limitations and future research

This study evaluated data arising from a unique circumstance, capturing a previously mine fire-affected community’s experiences during another wildfire event. While there has been limited research to date attending to communities dealing with multiple exposures to fire events, this study is timely given this circumstance is likely to become more common in future due to climate change. This cohort was established based on a population survey rather than a convenience sampling framework, which increases the representativeness of the sample.

There are also limitations to this research. All questionnaire items were self-reported, which renders them vulnerable to reporting bias. Only about half of the contacted participants responded to the follow-up survey, which could be a source of response bias. This analysis presented cross-sectional findings at the time of the 2019–20 Black Summer wildfires and longitudinal associations were not evaluated. The Mental Health and Wellbeing Survey was also not designed to evaluate the dynamic flow of symptoms (e.g., how symptoms were activated during the Black Summer fires), therefore this study cannot evaluate the causal associations between different symptoms.

The HHS will continue following up with the cohort to investigate the persistence of somatic symptoms and posttraumatic stress over time. Under the looming threat of climate change, future studies are needed to better understand the impact of other climate disasters (e.g., floods and cyclones) and the effectiveness of different clinical interventions. Given there are a number of efficacious treatments for PTSS/PTSD [38], clinical interventions may also have the potential to prevent chronic distress and somatic symptoms and this area needs more research [12]. Lastly, qualitative research exploring individuals’ lived experiences is needed to better understand the impacts of trauma and multiple losses from wildfires, and of living within the context of repeating wildfire events.

### Implications

Given the relationship between trauma, psychological distress, and somatic symptoms demonstrated both here and in previous research, it is essential that healthcare providers and public health authorities supporting communities affected by climate disasters take into account the way these conditions are interconnected. Further investigations of the effectiveness of PTSS treatment in ameliorating associated physical health issues are warranted. A climate change public health policy response, with increased investment and research into better supporting those affected, is urgently needed.

### Supplementary Information

**Additional file 1. **

## Data Availability

The dataset analysed in the current study is not publicly available as it is owned by the Victorian Department of Health and authors do not have the authority to share or distribute the third-party data. However, it is available from the corresponding author on reasonable request with approval from the Department.
