# Supplementary material for: Somatic symptoms, psychological distress and trauma after disasters: lessons from the 2014 Hazelwood mine fire and 2019–20 Black Summer bushfires
Source: BMC Public Health. 2023 Aug 18;23:1573. doi: 10.1186/s12889-023-16501-1 (PMC10436633; doi:10.1186/s12889-023-16501-1)
Supplement: Supplementary file 1 — Additional file 1. [file 12889_2023_16501_MOESM1_ESM.docx]

# Supplementary Material


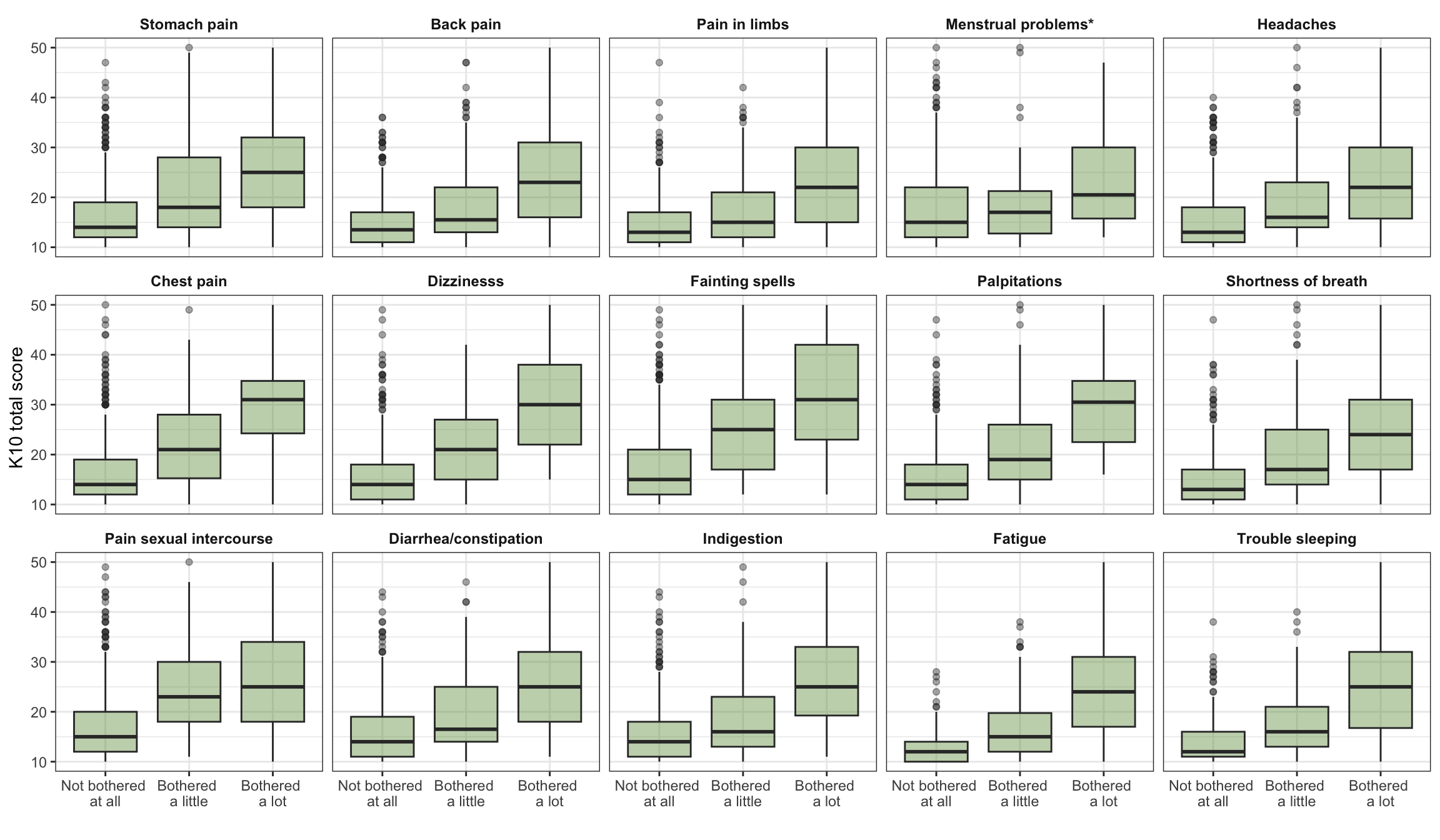


Figure S1. Box plotted distributions of K10 total scores for each PHQ-15 item

*Note.* *Females only.


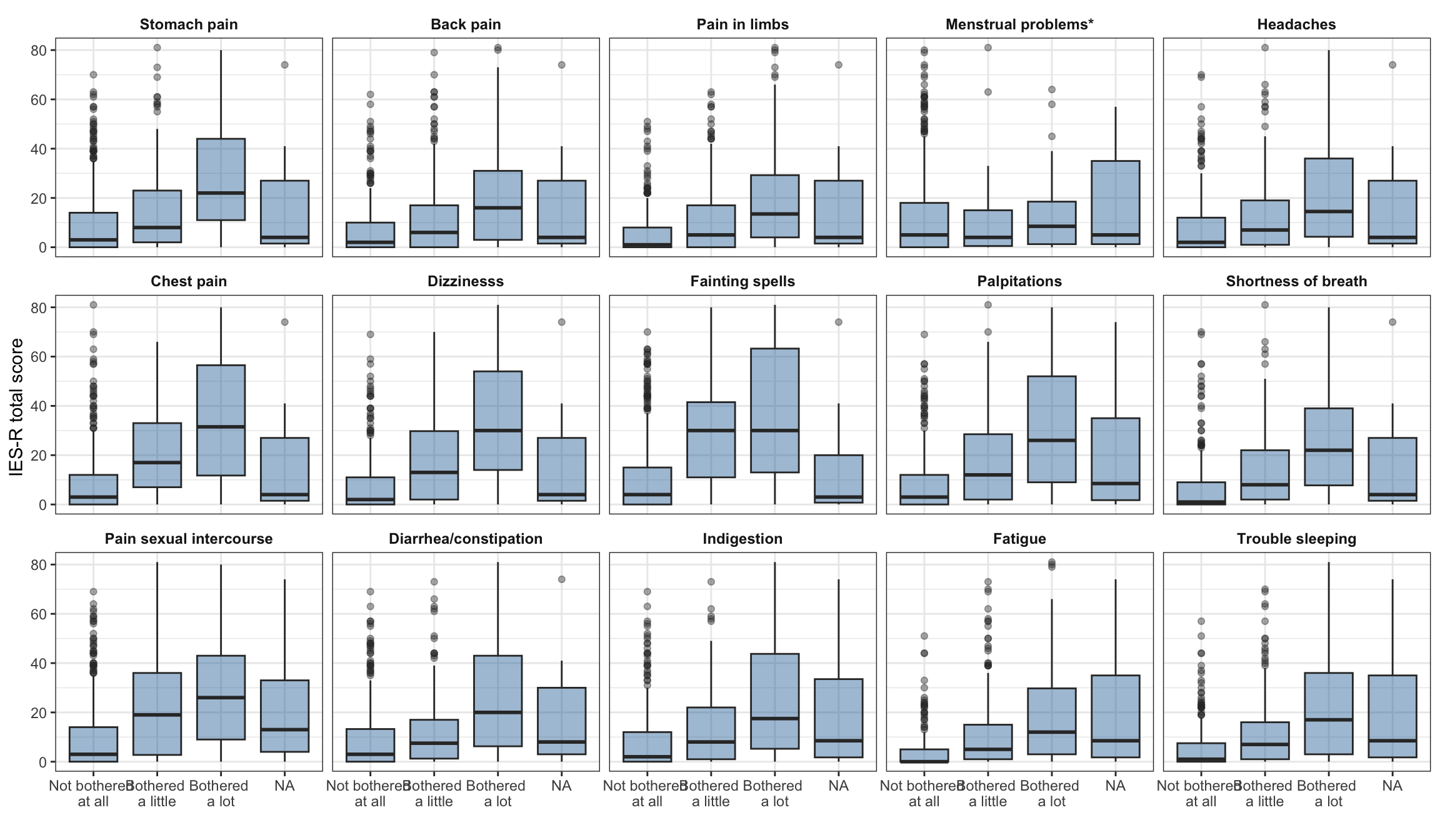


Figure S2. Box plotted distributions of IES-R total scores for each PHQ-15 item

*Note.* *Females only.

Figure S3. Pairwise polychoric correlations between individual items of the PHQ-15, K10, and IES-R


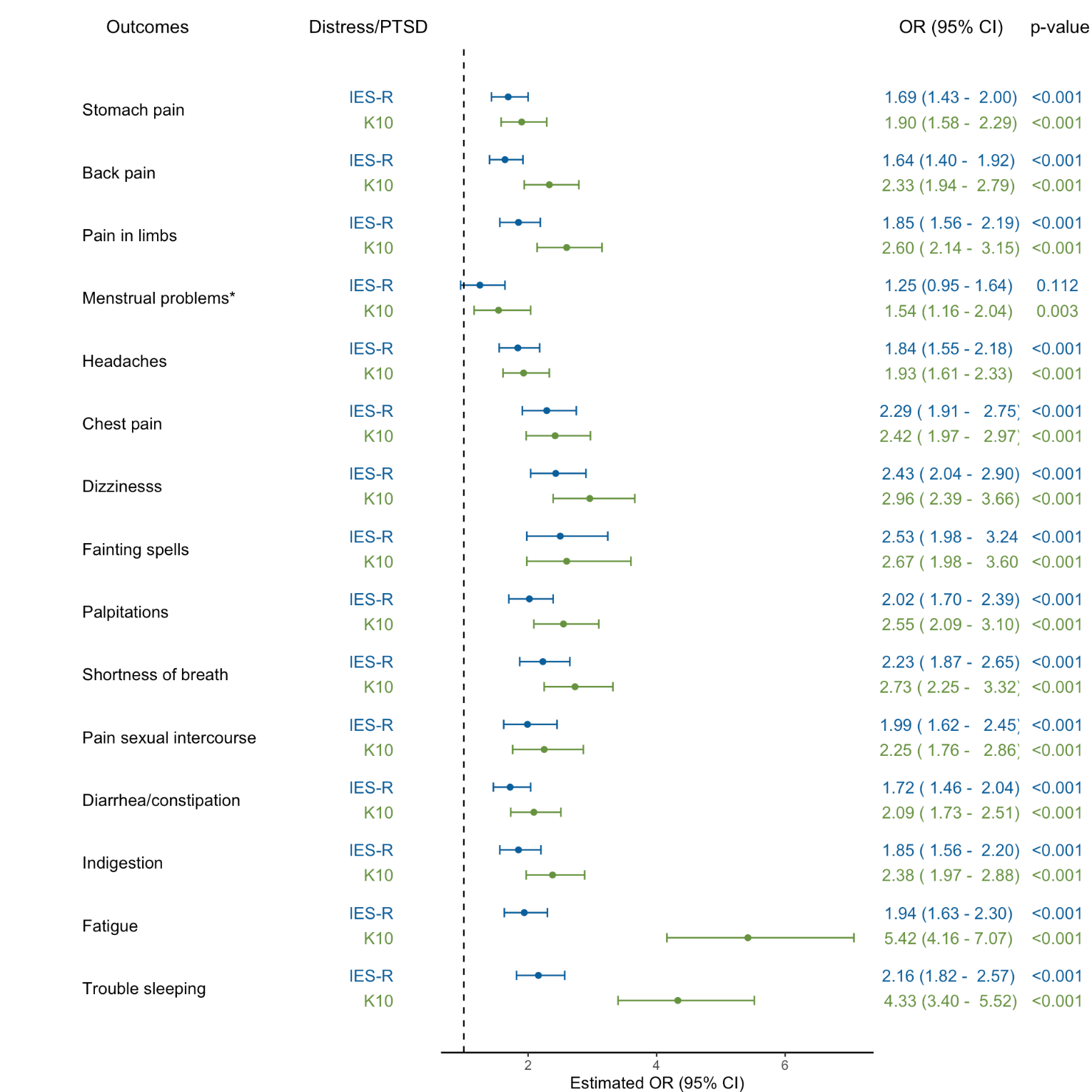


Figure S4. Estimated OR (95%CI) for individual somatic symptoms associated with a one standard deviation increase in IES-R and K10 scores

*Note.* For each PHQ-15 item, two imputed ordinal logistic regression models were used including IES-R or K10 separately as risk factors and controlling for confounders including age, gender (*except for menstrual problems evaluated in females only), education, employment, smoking status, diagnosed mental health conditions, and diagnosed physical health conditions.


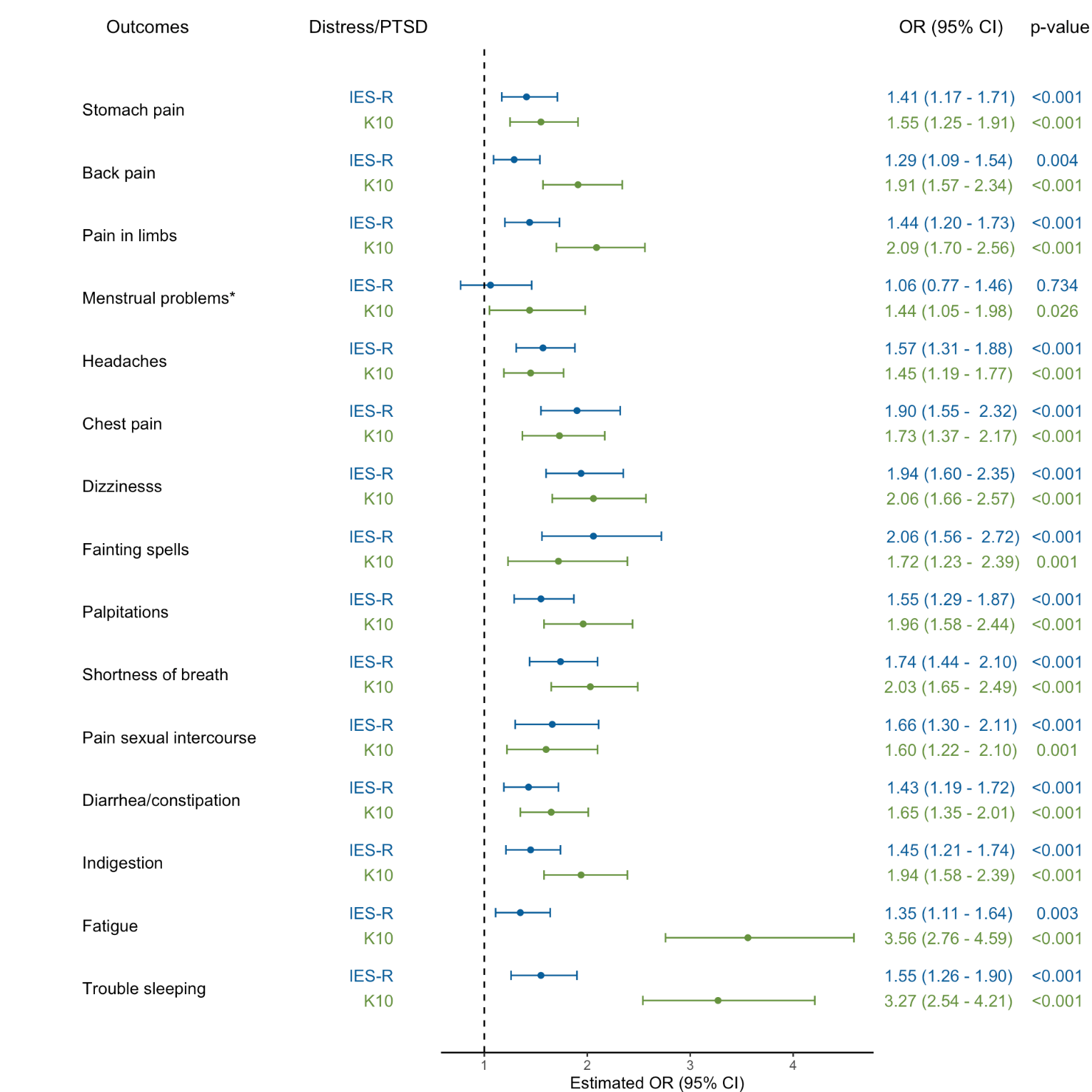


Figure S5. Estimated OR (95% CI) for individual somatic symptoms associated with a one standard deviation increase in IES-R and K10 scores (Q1 `feeling fatigued’ removed)

*Note.* For each PHQ-15 item, an imputed ordinal logistic regression model was used including both IES-R and K10 as risk factors and controlling for confounders including age, gender (*except for menstrual problems evaluated in females only), education, employment, smoking status, diagnosed mental health conditions, and diagnosed physical health conditions.

Table S1. Results from linear regression models with PHQ-15 total scores as the outcome variable

|  | **K10 and IES-R modelled separately** | |  | **K10 and IESR modelled together** | |
| --- | --- | --- | --- | --- | --- |
|  | **Coeff (95% CI)** | **p-value** |  | **Coeff (95% CI)** | **p-value** |
| **K10** | 4.13 (3.76 - 4.50) | <0.001 |  | 3.25 (2.85 - 3.65) | <0.001 |
| **IES-R** | 3.07 (2.70 - 3.45) | <0.001 |  | 1.67 (1.29 - 2.05) | <0.001 |

*Note.* Estimated coefficients (95% CI) represent changes in PHQ-15 total score associated with a one standard deviation increase in IES-R and K10 scores, estimated from imputed linear regression models controlling for confounders including age, gender, education, employment, smoking status, diagnosed mental health conditions, and diagnosed physical health conditions.

Table S2. Results from linear regression models with PHQ-15 total scores as the outcome variable and high correlation items removed

|  | **K10 and IES-R modelled separately** | |  | **K10 and IES-R modelled together** | |
| --- | --- | --- | --- | --- | --- |
|  | **Coeff (95% CI)** | **p-value** |  | **Coeff (95% CI)** | **p-value** |
| **K10** | 3.31 (2.93 - 3.69) | <0.001 |  | 2.40 (2.00 - 2.79) | <0.001 |
| **IES-R** | 2.82 (2.46 - 3.19) | <0.001 |  | 1.83 (1.46 - 2.21) | <0.001 |

*Note.* Estimated coefficients (95% CI) represent changes in PHQ-15 total score (Q14 `fatigue’ and Q15 ‘trouble sleeping’ removed) associated with a one standard deviation increase in IES-R and K10 scores (Q1 ‘feeling fatigued’ removed), estimated from imputed linear regression models controlling for confounders including age, gender, education, employment, smoking status, diagnosed mental health conditions, and diagnosed physical health conditions.
